# Judicial improvement, market integration, and export technical complexity-a quasi-natural experiments based on the creation of circuit courts

**DOI:** 10.1371/journal.pone.0296442

**Published:** 2024-03-15

**Authors:** Xin Gao, Shuo Kong

**Affiliations:** Anhui University of Finance and Economics, Bengbu, Anhui, China; Lingnan University, The University of HongKong, HONG KONG

## Abstract

Judicial improvement and trade structure optimization are important elements in achieving a high level of openness. This paper develops a theoretical framework on the impact of local protectionism on market integration and export technical complexity, and then empirically analyses the effect of judicial improvement on the technical complexity of urban product exports and its mechanism of action based on a quasi-natural experiment with the establishment of circuit courts. The study finds that the establishment of circuit courts significantly increases the technical complexity of product exports, and the findings remain valid after a series of robustness tests. Further analysis shows that the technology enhancement effect of the establishment of circuit courts is more pronounced in the group of cities with higher administrative levels, better location advantages, and less advanced rule of law. Mechanism Analysis shows that a superior institutional environment can have a two-sided effect on different types of firms. Overall, however, the productivity-enhancing effect of low-productivity firms exiting the market is lower than the productivity-reducing effect of higher-productivity firms’ increased compliance costs, which in turn weakens the positive promotional effect of circuit courts. The above theoretical and empirical findings provide policy implications for maximizing the technology enhancement effect of the establishment of circuit courts on product exports, thereby promoting a high level of openness in China.

## I. Introduction

In the face of the world’s unprecedented changes in the past century, General Secretary Xi Jinping’s keynote speech at the Boao Forum for Asia clearly emphasized that "China’s open door will not be closed, but will only be opened wider and wider". The report of the 20th Party Congress once again emphasizes the development layout of "adhering to a high level of opening up to the outside world, accelerating the construction of a new development pattern with the domestic circulation as the mainstay and the dual domestic and international circulation promoting each other". Adhering to a high level of openness will be a strategic choice to accelerate the construction of the "double cycle" and the construction of a strong trade country. Since the inception of the reform and opening-up policy, China’s foreign trade has witnessed a remarkable surge. The total trade volume has escalated from an initial $20.64 billion at the onset of reform and opening up to an impressive $63 trillion in 2022, securing its position as the world’s leading goods trading nation for six consecutive years. Notably, export volume has soared from $9.75 billion during the early stages of reform and opening up to an astounding $35.9 trillion, with "Made in China" captivating global markets. From the perspective of trade "quantity," China undoubtedly stands as a major trading nation. However, when scrutinized through the lens of trade "quality," it remains in a subordinate position within the international trade division of labor compared to other world trade powers. Therefore, during this crucial period of China’s high-quality economic transformation, the imperative of elevating the technological complexity of exported products to shift foreign trade from the growth of "quantity" to the development of "quality" holds significant importance. This shift is essential for achieving China’s dominant position in the global value chain, breaking free from the constraints of mid-to-low-end production, and realizing the aspirations of becoming a trade powerhouse aligned with high-quality economic development.

Hausmann et al. [1] define export technological complexity as a comprehensive indicator measuring the technological content and productivity of a country’s exported products. A higher value indicates a more elevated position within the global value chain [2]. The increase in export technological complexity falls within the technological domain. Apart from autonomous innovation by enterprises [3], competitive innovation [4], judicial perfection [5], and market integration [6] are effective pathways to enhance the technological complexity of exported products. The improvement of local judicial quality is crucial for ensuring efficient flow of technological elements and achieving high-quality integration of the domestic market. It is also a significant attempt to maintain enduring competitiveness in China’s export trade during the unprecedented transformation. Since the founding of the People’s Republic of China, the judicial system has progressively developed, but the quality of local judicial systems still exhibits deficiencies [7]. Local governments excessively rely on expanding administrative powers to intervene and regulate the market, leading to severe market segmentation while granting "special treatment" to local enterprises.

To curb the excessive expansion of local administrative powers and alleviate local protectionism, in October 2014, the Fourth Plenary Session of the 18th Central Committee of the Communist Party of China reviewed and passed a major decision on advancing the rule of law. The plenary session highlighted the optimization of judicial power allocation, promoted the pilot system reform of separating the powers of adjudication and enforcement, and established circuit courts under the Supreme People’s Court to primarily handle significant administrative and civil-commercial cases across administrative regions. The judgments, rulings, and decisions made by these circuit courts represent those of the Supreme People’s Court. In January 2015, the First and Second Circuit Courts were established in Shenzhen and Shenyang, respectively, covering the provinces of Guangdong, Guangxi, Hunan, Hainan, as well as Heilongjiang, Jilin, and Liaoning. In December 2016, the Third to Sixth Circuit Courts were set up in Nanjing, Zhengzhou, Chongqing, and Xi’an, respectively. At this point, the jurisdiction of the six circuit courts covered all regions nationwide except for Hong Kong, Macao, Taiwan, and the areas directly managed by the Supreme People’s Court, which include Beijing, Tianjin, Shandong, Hebei and Inner Mongolia.

The establishment of the circuit courts by the Supreme People’s Court is a significant strategic deployment determined at the Fourth Plenary Session of the 18th Central Committee of the Communist Party of China. It is a crucial measure to deepen the reform of the judicial system and build a socialist country ruled by law. This initiative provides a solid foundation for preventing judicial localization, enhancing the impartiality of local judicial systems, and offers an opportunity to observe the impact of judicial improvements on the enhancement of export technological complexity. In this context, this paper takes the establishment of the circuit courts by the Supreme People’s Court in 2015 as a quasi-natural experiment. It employs a multi-period Difference-in-Differences (DID) approach to assess the role of local judicial improvements in promoting the enhancement of export technological complexity. The study also empirically examines the interaction effects of judicial perfection and market integration on export technological complexity.

Compared with established studies, the marginal contribution of this paper is divided into three main aspects: first, it provides a reasonable quantification of judicial improvement. This paper uses the establishment of China’s circuit courts in 2015 to portray local judicial improvement, this indicator represents a key measure in the judicial reform process, making this paper’s research more representative and reasonable. Second, drawing on the theoretical analysis framework of Melitz [8] and Lv et al. [9], the theoretical analysis is anchored from the firm level to the city level, and the effect of judicial improvement on the technical complexity of product exports at the city level is explored from both theoretical and empirical perspectives, while the mechanism of the action of the institutional environment as a moderating variable and the effect of the influence are examined. Third, as a developing country with a legal and economic system in transition, the economic link between the judicial system and international trade is worthy of in-depth exploration, and the uniqueness of its research perspective is instructive for the construction of legal and economic institutions in transition economies, including China.

The remainder of the paper is organized as follows: Part II provides a review of the relevant literature. Part III details the institutional background and theoretical mechanisms, and presents the relevant theoretical hypotheses. Part IV presents the relevant data selection and research methodology. Part V presents the results of the empirical analysis; and finally, Part VI presents the conclusions and policy recommendations.

## II. Review of the literature

The research results related to this paper fall into three main categories: firstly, research on the economic effects of domestic market integration and the effects of export trade; secondly, research on the effects of judicial improvement on domestic market integration and economic development; and thirdly, research on the factors influencing the technological complexity of exports.

### (i) Research related to domestic market integration and export trade

Market integration, as opposed to local protection and market segmentation, is a process of building a unified and open market, gradually breaking down and eliminating local protectionism as well as enhancing the tightness of inter-regional markets. In promoting the free flow of goods and factors, market integration helps to optimize the allocation of resources and reduce transaction costs. Therefore, the cost effect and rational allocation of resources resulting from market integration have considerable economic effects as well as export trade effects. Its economic effects are mainly reflected in economic growth, the increased innovation capacity of enterprises, and improved resource allocation efficiency [10–12], and export trade effects are mainly reflected in the decrease of export scale and the increase of export technical complexity [6, 13].

Based on the national level, Poncet [14] combined with China’s trade flows at the inter-provincial level and found that between 1987 and 1997, local protectionism segmented China into different provincial markets, with each provincial market participating freely in international trade, significantly increasing provincial participation in international trade. With the increasing abundance of microdata, some scholars have extended their research to the firm level. Based on the firm level, market integration affects firms’ export behavior through market competition mechanisms. Hou et al. [15] find that market integration lowers market entry barriers for heterogeneous firms and stimulates market competition, which in turn affects firms’ product export choices. Low market integration tends to attract efficient firms to operate in the domestic market and stimulate inefficient firms to export [16]; moreover, this choice of firm export behavior relies mainly on cost effects. Low market integration implies that it is more costly for firms to enter the domestic market, and more difficult it is for firms to enter the domestic market and instead enter the international market [17]; however, in addition to the cost effectiveness, the rational allocation of factor resources resulting from high market integration will facilitate the expansion of exports of high-efficiency firms [18]. Another part of the literature focuses more on the effects of market integration on the technical level of exports. Mao [19] find that regional market integration would highlight regional comparative advantages, optimize resource allocation, promote specialized division of labor, and thus enhance the technological level of regional product exports, ultimately increasing the value added of enterprises’ exports [9, 20]. Lei and Lang [6] further find that the limitations of threshold variables such as human capital and innovation constraints make market integration regionally heterogeneous in terms of the increase in technological sophistication of product exports.

### (ii) Studies of the effects of judicial improvements on market integration

In the course of China’s judicial development, local governments have, to a large extent, the ability to interfere with local judicial power, which has led to local protectionism. Local protectionism has fostered judicial corruption and impeded judicial progress and the improvement of judicial quality [7]. Local protectionism has led to the inability of local judicial power to be exercised independently and impartially in China, and the effective implementation of judicial power has been severely constrained [21], while local protectionism, in providing ’special treatment’ to favored local enterprises, has restricted normal and orderly market competition among enterprises, exacerbated regional trade barriers and ultimately results in market fragmentation [22, 23]. Deepening market segmentation will further restrict corporate investment, with heterogeneity in industry investment and underinvestment in some sectors [24, 25]. Donaldson [26] further links local protectionism, market integration, and business returns, and finds that local protectionism leads to market segmentation that reduces firm returns. In China, local protectionism is a by-product of China’s ’jousting’ political ladder [27] and has severely hampered China’s economic integration [28]. At the same time, local protectionism has profoundly affected the development of regional industrial specialization in China, distorting the formation of industrial clusters [29] and causing severe welfare losses. Barwick et al. [30] quantified the impact of local protectionism by developing a market equilibrium model. The research findings reveal that local protectionism has resulted in severe distortions in decision-making, with preferential actions by local governments causing significant consumer welfare losses. Bao and Huang [31] explored the impact of artificial intelligence on gender equality, suggesting that the neutral behavior of artificial intelligence can contribute to societal equality. Consequently, the neutrality in local government behavior induced by judicial perfection can mitigate the degree of market segmentation, thereby fostering market integration. Therefore, to enhance and improve the quality of local justice and mitigate local protectionism, in 2015 the Supreme People’s Court of China embarked on the establishment of local circuit courts, which proved that an independent judicial system can significantly reduce local protection and promote market integration.

An effective judicial system is a fundamental cornerstone of a country’s economic prosperity [32]. A sound judicial system is conducive to good economic performance, with judicial improvements as well as constitutional review contributing to a country’s economic freedom [33] and aiding domestic trade as well as corporate exports [34]. Chen and Li [35] and Cao et al. [36] used the 2008 exchange of provincial high court presidents across China to reflect local judicial improvements. They found that judicial improvements not only reduced market segmentation but also promoted economic growth. Judicial administrative nation inhibits reasonable competition among firms, hinders business development, and limits economic growth [37]. The establishment of circuit courts has effectively de-localized the judiciary, improved the quality of local justice, provided effective protection for non-local businesses’ intellectual property rights, etc., which in turn has provided an incentive for businesses to invest more [38].

While research with a judicial improvement perspective is relatively well established, studies focusing on circuit courts are still in the minority. It is worth emphasizing, however, that China’s legal system is highly unified, established by the central government, and implemented specifically at the local level, and this paper focuses not on the quality of the judicial system at the national level, but on the details of local implementation and its impact on the domestic market integration dimension. Focusing on the implementation of the establishment of circuit courts in 2015, this paper explores the export trade effects of judicial improvements in terms of their theoretical orientation to alleviate local protectionism and promote domestic market integration and enhance the technical complexity of product exports.

### (iii) Studies of the factors influencing the technical complexity of exports

High-quality and sustainable economic development requires not only the expansion of trade but also the optimization of trade structures, which is reflected in the increase in the technical complexity of exports [39]. Chen et al. [40] investigated the dynamic correlation between market connectivity and risk spillover. Market connectivity in stock markets reflects the information efficiency of capital markets, where the lack of regulation leads to pronounced stock market volatility, thereby further amplifying risk transmission. Consequently, judicial perfection plays a crucial role in market connectivity, contributing significantly to fostering market integration and, consequently, enhancing export technological complexity. Related studies on the enhancement of export technological complexity have focused on trade liberalization [41], the digital economy [42], the judicial system [43–46], among other perspectives, while one of them is to examine the effect of the judicial improvements on the technical complexity of exports, using intellectual property protection, administrative approval system reform, and institutional quality as a starting point.

The influence of the judicial system on export technical complexity has been largely agreed upon by domestic and foreign scholars, who all agree that a well-developed judicial system can drive export technical complexity. Meon and Sekkat [43] find that a well-developed rule of law system promotes a country’s export technical complexity by reducing the transaction and production costs of products. However, regional heterogeneity makes the positive effect unstable, but the overall positive trend remains unchanged [44]; there is also a significant positive trend of regional intellectual property protection and export technology sophistication [45, 47], but the stronger positive correlation shows industry heterogeneity, with technology-intensive industries having the most significant intellectual property protection effect on product export technology enhancement [48]; with the extension of the research perspective, it is found that the promotion of the administrative approval system reform reduces the uncertainty faced by enterprises in the transaction process, thus promoting the technical complexity of firms’ exports [46]. At the same time, there is a scale heterogeneity in the increase in the technical complexity of exports by enterprises through the establishment of administrative approval centers, with a more significant increase in the technical complexity of exports by small-scale enterprises and general trading enterprises [49].

As outlined above, existing literature on the influence of judicial perfection, administrative approval systems, and the export trade effects of domestic market integration has reached a relatively mature stage. However, there remains a relative scarcity of research that comprehensively examines export technological complexity from the combined perspectives of judicial perfection and domestic market integration. Building upon this foundation, this paper utilizes a multi-period Difference-in-Differences (DID) model, grounded in the quasi-natural experiment of the establishment of circuit courts, to investigate the impact of judicial perfection on export technological complexity. Additionally, the study delves into the interaction effects of judicial perfection and domestic market integration on the complexities of exported technologies.

## III. Institutional background and theoretical mechanisms

### (i) Institutional background

Since the reform and opening up of China, the judicial system has been beneficially improved, but the fiscal decentralization and GDP-oriented economic development bidding tournament have incentivized local governments to protect local enterprises, resulting in local protectionism [50]. An important way in which local governments exercise local protectionism is by influencing local courts. In principle, courts are supposed to be independent organizations that can fairly resolve conflicts between local and external companies. In practice, because local courts are highly dependent on local officials for personnel and financial decisions, local governments require judges to favor local defendants over external plaintiffs, especially when the local defendants have significant economic or political connections. A similar theme often appears on the Chinese Internet, where it is claimed that many large and influential companies are simply "invincible" in local courts [51].

To alleviate local protectionism, get rid of the close ties between local courts and local governments, and systematically enhance judicial credibility, the Supreme People’s Court has established local circuit courts. The circuit courts have broken the situation in which judicial power was excessively held back by administrative power in several ways. Firstly, the circuit courts have broken the problem of overlapping judicial and administrative powers. The circuit courts involve the unified management of people, finances, and materials by the Supreme People’s Court and are independent of local governments, effectively curbing the localization of justice and improving the quality of justice; secondly, in order to maintain the independence of judges, the presiding judges of the circuit courts are rotated every two years, and the results of their trials are the joint responsibility of the collegiate court and the presiding judges, i.e., "let the judge decide and let the judge be responsible" [51]; finally, in terms of trial effectiveness, the trial effectiveness of the circuit court is equivalent to that of the Supreme People’s Court and its trial level is higher than that of the Provincial High People’s Court, enabling it to effectively supervise the local courts at the provincial and sub-provincial levels.

After the reform, local governments no longer have much influence over local courts, thus greatly enhancing their independence and improving the quality of local justice, which has become a milestone in the history of China’s legal development. Therefore, further research on the judicial improvements resulting from the establishment of circuit courts has important theoretical implications and practical significance, and some scholars have conducted extensive qualitative analyses of the establishment of circuit courts in China [52, 53], but few of these studies have linked judicial improvements to trade, especially to trade structure. As Zhang and Ginsburg [54] conclude, the establishment of circuit courts has raised the quality of justice in China to an "unprecedented level", and the effect of this "unprecedented level" of judicial quality on the structure of trade, and more importantly, on the technological sophistication of exports, deserves to be explored in depth, in order to help better understand the deeper implications of judicial improvement in the context of the policy of a strong trading nation.

### (ii) Theoretical mechanisms

The notable enhancement of judicial quality has been shown to significantly promote the upgrading of export product quality [55], contributing to the elevation of export product technological complexity. A high level of judicial quality can enhance the fairness and predictability of market transactions, improve contractual environments [56] and safeguard intellectual property rights, thereby boosting innovation capabilities and elevating the quality of exported products.

Firstly, judicial improvement, such as strengthening the protection of intellectual property rights, can effectively stimulate technological innovation for enterprises, especially those in the manufacturing sector with "high, precision, and cutting-edge" technologies [57], consequently raising the technological complexity of exported products. Intellectual property protection not only increases imitation costs and incentivizes innovation [58] but also, from a supply perspective, can enhance a company’s property advantages, promote innovation, and elevate the upstream position of a company’s exports in the global value chain [59]. On one hand, intellectual property protection can effectively restrain imitation activities, reduce the innovation risks for enterprises, and encourage increased research and development investment in products, processes, and technology, fostering long-term technological progress. On the other hand, strengthening intellectual property protection in the home country can significantly increase the scale of foreign direct investment by enterprises [60], facilitating the undertaking of higher-complexity production processes. This, in turn, promotes the improvement of technological assets and innovation levels, creating a competitive advantage and enhancing the quality and market attractiveness of company products.

Secondly, in cities where administrative approval centers have been established, there has been a significant increase in the export technological complexity of manufacturing enterprises [61]. The cost-effectiveness and innovation incentives generated by the "one-stop" approval centers optimize the functions of the Chinese government, allowing the innovation potential of market entities to be unleashed. This accelerates the gathering of innovative elements, increases enterprise innovation activities, shifts product production towards high-end specialization, detaching from low-end production segments of the value chain, and consequently enhances export technological complexity. Furthermore, ongoing policies aimed at improving the judicial environment, such as the establishment of intellectual property demonstration cities [62], the establishment of municipal patent agencies [63], the signing of the "patent examination expressway" agreement [64], and the establishment of circuit courts [5], continue to promote the improvement of export product quality, facilitate industrial structural upgrades, and thereby raise the technological complexity of exported products.

Based on these observations, this paper proposes:

Hypothesis 1: Judicial improvement has a significant promoting effect on the technological complexity of exported products.

Judicial independence is not only a prerequisite and foundation for judicial fairness but also a primary symbol of a modern rule-of-law state and a crucial element of contemporary constitutional governance. The pursuit of independent judicial departments and the empowerment of these departments with autonomous adjudicative authority represent fundamental goals in China’s legal construction. The enhancement of the independence of local judicial departments serves to effectively balance governmental powers, mitigate regional protectionism, promote market integration, and induce credible commitments from local governments to protect private property and contractual freedoms. This is conducive to achieving high-quality economic development [35]. Furthermore, strengthening constraints on bureaucratic behavior can enhance the market value of enterprises [65], fostering long-term economic growth. Thus, judicial independence holds not only significant constitutional implications but also substantial economic value.

The establishment of circuit courts is a crucial step in China’s ongoing efforts to perfect its judicial system. It not only enhances the adjudicative capabilities of local judicial systems but also reduces local administrative interference in judicial affairs, breaking down market segmentation. This has notably spurred the expansion of investments by enterprises with high technological complexity within the circuit areas, leading to an increase in overall factor productivity. High-productivity enterprises tend to have lower production costs and produce higher-quality export products [66]. However, against the backdrop of an increasingly perfected judicial system in China, while market integration has improved, additional investments in judicial services have increased manufacturing production costs, squeezing research and development (R&D) funding. Due to heterogeneity among enterprises, this has resulted in increased uncertainty regarding the technological complexity of exported products.

On one hand, a favorable institutional environment facilitates the integration of factors and product markets, alleviating the negative impact of regional protectionism to some extent. This aids in the growth of manufacturing productivity [67], prompting upgrades in export product quality and enhancing the technological complexity of exported products. On the other hand, significant institutional differences between regions can act as barriers for enterprises entering markets, significantly raising entry barriers for low-productivity enterprises, leading to increased rent-seeking costs and crowding out R&D capital, thereby lowering enterprise productivity. Simultaneously, for high-productivity enterprises, institutional improvements may not have a significant impact, but the tightening of judicial constraints could increase compliance costs, to some extent, reducing their productivity. In the long term, the exit of low-productivity enterprises results in an overall increase in urban enterprise productivity [8], known as the resource allocation effect.

Since the 18th National Congress of the Communist Party of China, the country has been actively advancing supply-side structural reforms, accelerating economic structural adjustments, and promoting transformational upgrades. The optimization of industrial structures has led to a continuous increase in the overall factor productivity of various industries. Therefore, overall, the positive impact of judicial improvement and increased market integration on the productivity enhancement resulting from the exit of low-productivity enterprises is lower than the negative impact of increased compliance costs on the productivity decrease for high-productivity enterprises.

Based on these observations, this paper proposes:

Hypothesis 2: The productivity enhancement effect from the exit of low-productivity enterprises is lower than the productivity decrease effect from increased compliance costs for high-productivity enterprises, thus weakening the proactive role of circuit courts.

## IV. Study design

### (i) Model setting

This paper uses the six batches of circuit court policy pilots listed in 2015 and 2016 respectively as exogenous shocks to the export trade effects of cities and constructs a multi-temporal DID benchmark regression model with cities within the provinces covered by circuit courts as the treatment group and other cities as the control group. The export trade effect of the circuit court policy pilot is examined from the perspective of the technical complexity of exports. The following regression models are constructed.


$${ESI}_{it}=\alpha +\beta_{1}{ICTs}_{it}+\beta_{2}X_{it}+\mu_{t}+\gamma_{i}+\varepsilon_{it}$$ (1)

Where the subscripts *i* and *t* denote city and year respectively; *ESI*_*it*_ denotes the export technical complexity of city *i* in year *t*; *ICTs*_*it*_ denotes a dummy variable for whether city *i* has a pilot circuit court in year *t*, taking a value of 1 if city *i* is a pilot city in year *t* and 0 otherwise. *X*_*it*_ denotes a set of control variables, namely regional GDP, foreign direct investment, level of financial development, degree of urbanization, industrial structure, the strength of environmental regulation, and infrastructure development; *γ*_*i*_ is a firm fixed effect; *μ*_*t*_ is a year fixed effect; *ε*_*it*_ is a random error term; and the estimated coefficient *β*_1_ denotes the effect of the pilot circuit court policy on the technical complexity of city exports.

### (ii) Selection of variables

#### (1) Explanatory variables

This paper extends the methodology developed by Hausmann et al. [1] for calculating export technological complexity to the urban level, conducting a comprehensive assessment of export technological complexity across various regions in China. The calculation formula is presented as follows:

$${Prody}_{kt}=\sum_{i}\frac{X_{ikt}/X_{it}}{\sum_{i}(X_{ikt}/X_{it})}{pergdp}_{it}$$ (2)

$${ESI}_{it}=\sum_{k}\frac{X_{ikt}}{X_{it}}{Prody}_{kt}$$ (3)

To begin, the calculation of the technological complexity index for industry *k*, denoted as *Prody*_*kt*_, is derived according to Eq (2). Here, *X*_*ikt*_ represents the export value of industry *k* in region *i* for the year *t*, *X*_*it*_ represents the total export value across all industries in region *i* for the same year, and *pergdp*_*it*_ signifies the real per capita Gross Domestic Product (GDP) for region *i* in the year *t*. Subsequently, Eq (3) is applied, using the proportions of exports from each industry in region *i* to the total exports across all industries in region *i* as weights. This weighted sum is then used to calculate the regional export technological complexity index, denoted as *ESI*_*it*_.

#### (2) Core explanatory variables

This paper treats the establishment of circuit courts as a quasi-natural experiment, and the policy effect of the urban pilot of circuit courts (*ICTs*_*it*_) is represented by the interaction term of the pilot city dummy variable (*pilot*) and the policy implementation time dummy variable (*time*). The first circuit court was inaugurated on 28 January 2015, the second circuit court was inaugurated on 31 January 2015, the third and fourth circuit courts were inaugurated on 28 December 2016, and the fifth and sixth circuit courts were inaugurated on 29 December 2016. Considering the time lag in the establishment and implementation of circuit courts, this paper sets the time points for the establishment and implementation of the six circuit courts as 2015 and 2017 respectively. Therefore, for the years 2015 and 2017, the cities designated as pilot cities for circuit court trials are assigned *ICTs*_*it*_ = 1 for the respective years and subsequent years, while the remaining cities are designated as *ICTs*_*it*_ = 0.

#### (3) Control variables

Considering that other factors at the city level may have an impact on the technical complexity of exports, the control variables in this paper include, **①** gross regional product (*GDP*); **②** foreign direct investment (*FDI*), measured by the amount of foreign capital used in the city in the current year; **③** financial development level (*finance*), measured by the ratio of the number of people employed in finance to the total number of people; **④** urbanization (*urban*), measured by the ratio of the number of non-agricultural population to the total population at the end of the year; **⑤** industrial structure (*industry*), measured by the proportion of value added in the secondary industry to GDP; **⑥** environmental regulation (*er*), measured by the frequency of environmental protection words and regulation data in each prefecture-level government work report; **⑦** infrastructure development (*infra*), measured by the sum of total postal services and total telecommunications services, and then taking the natural logarithm. The descriptive statistics of the variables are shown in Table 1.

**Table 1:** Descriptive statistics.

	Obs	Mean	SD	Min	Max
*ESI*	3696	15.1422	8.3098	1.6418	56.2594
*ICTs*	3696	0.0909	0.2875	0.0000	1.0000
*GDP*	3696	19.4183	28.5433	0.6033	326.7987
*FDI*	3696	5.2466	12.4224	0.0002	204.7531
*finance*	3696	4.0243	3.6664	0.4274	47.6670
*urban*	3696	0.3793	0.2226	-0.0850	2.7743
*infra*	3696	12.4738	1.0037	9.3917	16.5024
*industry*	3696	48.2879	10.3086	14.9500	85.9200
*er*	3696	0.2163	1.0288	0.0000	35.1005

### (iii) Data sources and processing

To establish a valid experimental and control group, the data period of this paper is 2005–2018, with the cities covered by the establishment of six batches of circuit courts as the experimental group. The technical complexity of exports at the city level is also measured through the China Customs database. In addition, the other city-level data in this paper are mainly obtained from the city-wide data of the China City Statistical Yearbook in previous years, and the missing values of individual samples are combined with local statistical yearbooks, statistical bulletins, or by using linear interpolation to complete the data. And then, the data on economic case completion rates by province in the analysis of heterogeneity in the rule of law dimension was obtained from the China Law Yearbook for various years. The data on corruption at the provincial level in the analysis of political and business environment heterogeneity was obtained from the China Procuratorial Yearbook and the work reports of the provincial and municipal people’s procuratorates and contains the number of cases of official crimes (corruption and bribery + dereliction of duty) in each province.

## V. Empirical analysis

### (i) Baseline regression

Table 2 reports the regression results for the effect of the establishment of circuit courts on the technical complexity of product exports at the city level. In particular, column (1) does not take into account control variables and city and year fixed effects, and its model estimation results indicate that the technical complexity of product exports is effectively increased in pilot cities compared to non-pilot cities and that the coefficient of impact is relatively large. Column (2), on the other hand, further excludes time and regional confounding factors and controls for city and year-fixed effects, leading to a consistent conclusion with only weak coefficient differences. The inclusion of city-level control variables in column (3) reveals significantly larger and more significant coefficient estimates for the core explanatory variables. Other things being equal, column (4) remains robust to the empirical results after further accounting for time and regional confounding factors in column (3), the regression coefficients for *ICT*_*s*_ are all significantly positive, indicating that the establishment of circuit courts significantly increases the technical complexity of product exports. In addition, regional GDP, foreign direct investment, financial development level, urbanization and infrastructure development all contribute to the technical complexity of product exports, in line with theoretical expectations, while a high share of secondary industries and stringent environmental regulations are not conducive to the technical complexity of product exports. As mentioned above, the establishment and promotion of circuit courts have, on the one hand, provided good policy support for the rule of law in the cities covered; on the other hand, it has increased local judicial independence, reduced local protectionism, lowered rent-seeking costs, squeezed out R&D funds, which in turn affected productivity and ultimately led to an increase in the technological complexity of exports.

**Table 2:** Baseline regression.

	*ESI*			
	(1)	(2)	(3)	(4)
*ICTs*	12.9377***	0.6475***	8.8046***	0.6281***
	(0.4252)	(0.2204)	(0.3576)	(0.1890)
*GDP*			0.1067***	0.0196***
			(0.0081)	(0.0042)
*FDI*			-0.1476***	0.0189**
			(0.0153)	(0.0077)
*finance*			0.2713***	0.3561***
			(0.0392)	(0.0484)
*urban*			0.8875*	0.5118
			(0.5311)	(0.3926)
*infra*			1.6870***	0.0056
			(0.1441)	(0.1117)
*industry*			-0.2101***	-0.1958***
			(0.0097)	(0.0091)
*er*			0.0486	-0.0141
			(0.0958)	(0.0141)
_*cons*	13.9660***	15.0833***	0.7085	22.3641***
	(0.1282)	(0.0451)	(1.7771)	(1.4671)
*City fixed effects*	*NO*	*YES*	*NO*	*YES*
*Year fixed effects*	*NO*	*YES*	*NO*	*YES*
*N*	3696	3696	3696	3696
adj. *R*^2^	0.200	0.930	0.485	0.947

Note: Robust standard errors are in brackets

*, **, and *** denote significance levels of 10%, 5%, and 1% respectively. Same as later tables if not otherwise stated.

It can be seen that, under the top-level design of the central government, the establishment of circuit courts, which were planned by local governments on their own in conjunction with the actual situation, has effectively broken down local protectionism and accumulated successful experience in promoting the construction of judicial independence at the national level.

### (ii) Analysis of parallel trends and dynamic effects

The key premise of using a multi-temporal DID model is that pilot and non-pilot cities need to maintain a consistent trend in the technological complexity of product exports prior to policy implementation, i.e., satisfy the parallel trend test assumption. Therefore, this paper prioritizes the verification that the results of the baseline regression are not caused by time effects, but rather by the establishment of the circuit court. As the timing of policy implementation varies across pilot cities, a particular year cannot simply be taken as the threshold for policy shocks, but rather the relative timing dummy variable for the establishment of circuit courts needs to be specified for different regions. In this paper, Eq (14) is constructed to conduct a parallel trend test as follows.


$${ESI}_{it}=\alpha +\beta_{1}{Before6}_{it}+\beta_{2}{Before5}_{it}{+\beta }_{3}{Before4}_{it}+\beta_{4}{Before3}_{it}+\beta_{5}{Before2}_{it}{+\beta_{6}{Before1}_{it}+\beta_{7}{Current}_{it}+\beta_{8}{After1}_{it}+\beta_{9}{After2}_{it}+\beta_{10}{After3}_{it}+\beta }_{11}X_{it}+\mu_{t}+\gamma_{i}+\varepsilon_{it}$$ (4)

Where the time dummy variables are the observations for the *n* years before, the current year, and the *n* years after each city was established as a pilot city. The dummy variables for non-pilot cities are all 0. Since the sample period of this paper is 2005–2018, and the year of policy implementation for the first batch of circuit court city pilots is 2015, the data for the six years before the implementation of the pilot policy are merged into period -6, and the data for the three years after the implementation of the pilot policy are merged into period 3. The rest of the variables have the same meaning as in (1), and the period when the policy was implemented is taken as the base period.

**Fig 1:**
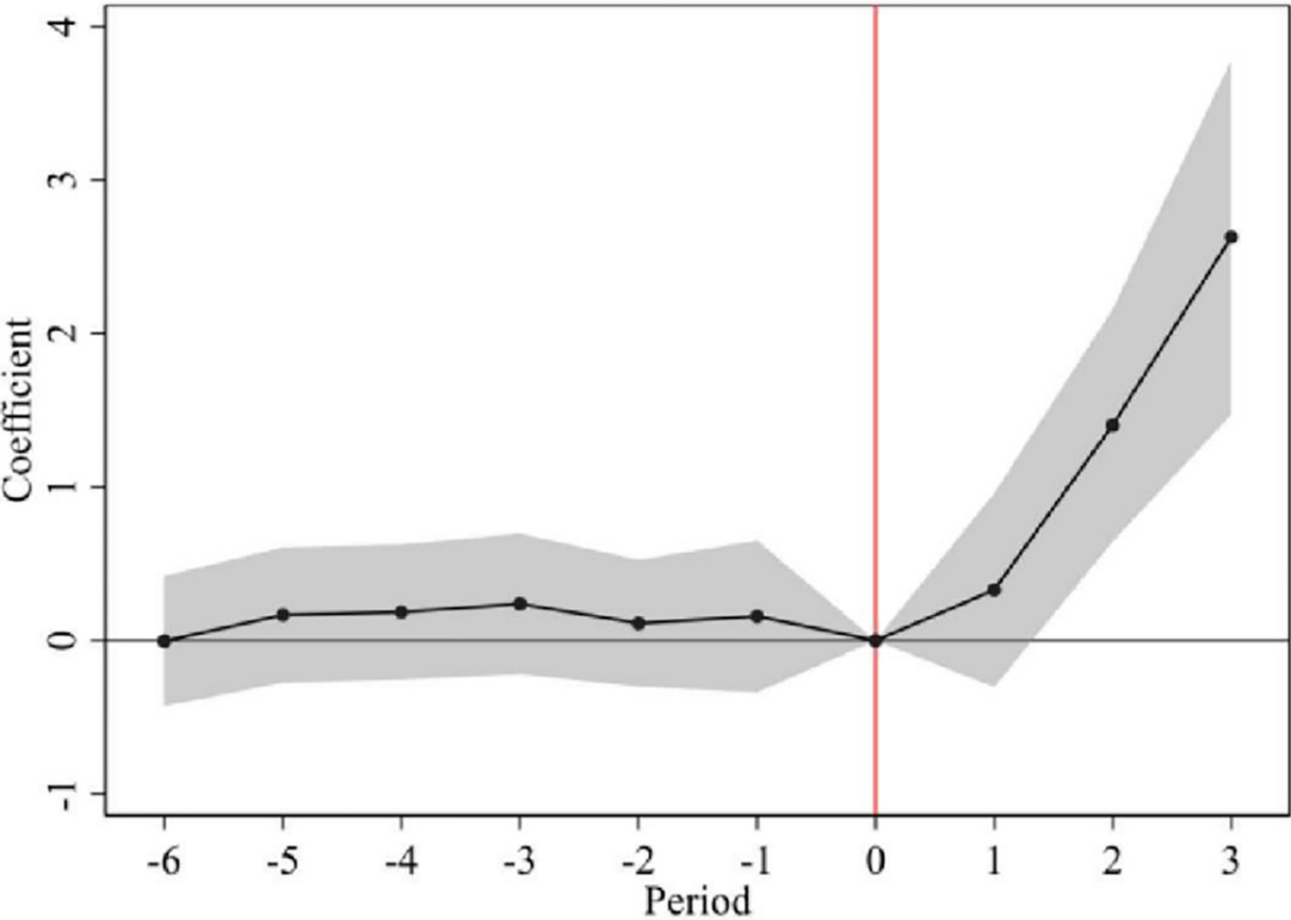
Parallel trend test.

The results are shown in Fig 1. The coefficients of the relative time dummy variables before the onset of the policy are all insignificant and small, indicating that there is no significant difference between the experimental and control groups in terms of export technical complexity before the onset of the policy, i.e., the establishment of the circuit court is consistent with the parallel trend hypothesis. In terms of the dynamic effect of the policy, the effect of the pilot policy was always significantly positive after the policy was implemented, and the coefficient of impact was significantly higher one year after the policy was implemented, and the effect deepened progressively over time. This suggests that there is a considerable product technology content upgrading effect of the policy implementation. Therefore, this paper concludes that the continuous improvement of the establishment of circuit courts can have the policy effect of promoting the upgrading of the technological sophistication of urban product exports. Overall, the results of the parallel trend test further support the findings of this paper.

### (iii) Placebo test

Although most of the city characteristics variables have been controlled for in this quasi-natural experiment, there may still be some unobserved city characteristics factors that could influence the assessment of circuit court establishment. To exclude unobservable factors from influencing the findings of this paper, a placebo test was conducted by generating a ’pseudo’ treatment group. As the ’pseudo’ treatment group is randomly generated, the establishment of a pseudo circuit court should not have a significant effect on the technical complexity of product exports and the estimated regression coefficients should be concentrated around 0.

**Fig 2:**
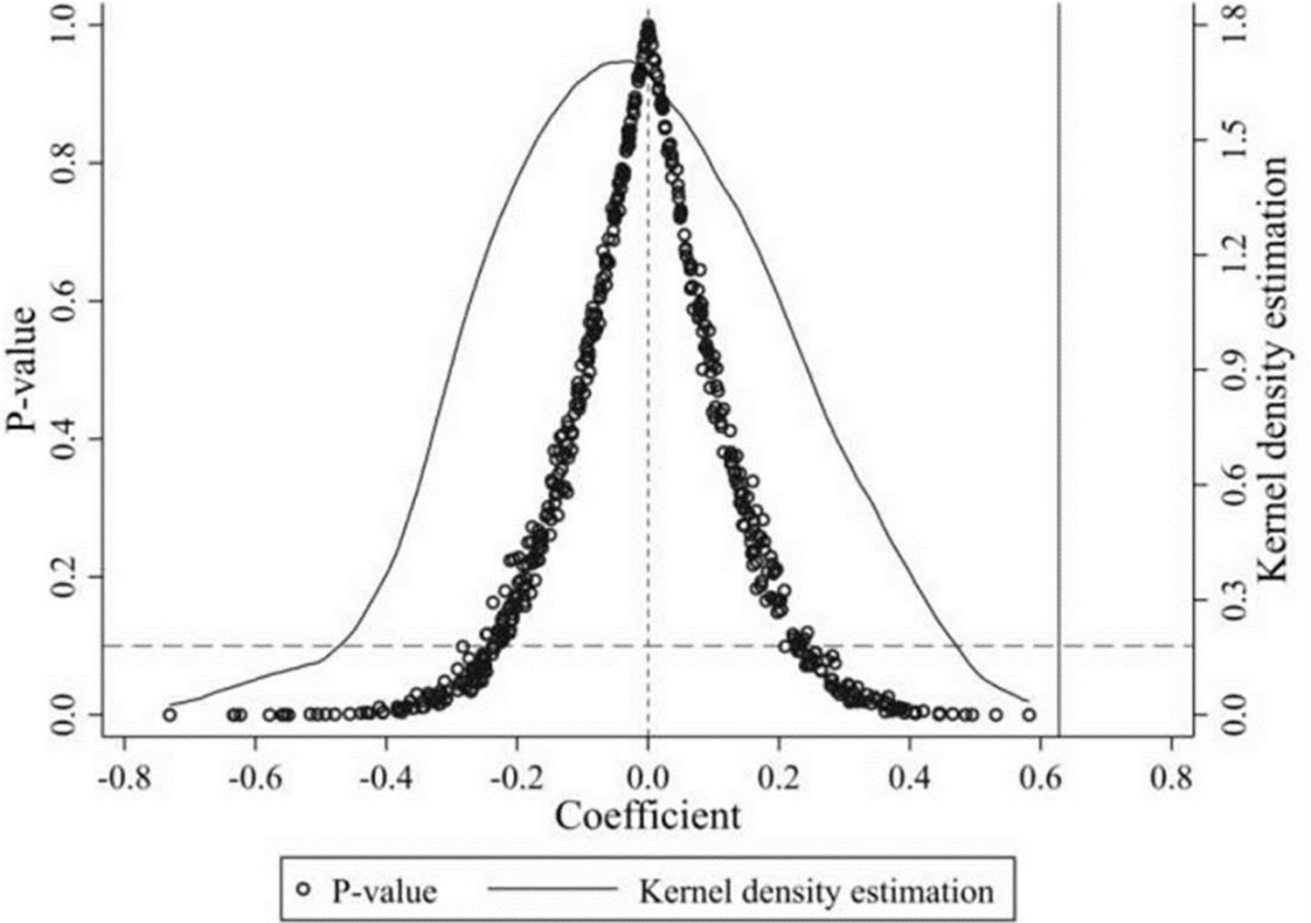
Placebo test.

Based on this, this paper constructs 500 random shocks of the pseudo-circuit establishment on 264 sample cities and presents the kernel density of the obtained 500 estimates of the pseudo-circuit establishment and their p-values in Fig 2. The results show that the mean values of the estimated coefficients of the policy implementation variables during the randomization process are mainly concentrated around 0 and have p-values mostly above 0.1, while the estimated coefficient of the actual policy is 0.6281 in column 4 of Table 2 lies in the region of small probability events in the kernel density plot, significantly different from the placebo test results. This suggests, to some extent, that the quantitative assessment results in this paper are not significantly influenced by unobservable factors and that the results are robust.

### (iv) Robustness analysis

The above baseline regression results and the multi-temporal DID test suggest that the establishment of circuit courts significantly increases the technical complexity of product exports. To exclude the influence of other confounding factors on the findings, this paper conducts a series of robustness tests in terms of removing outliers, propensity score matching double difference models, and excluding another environmental policy confounding, respectively.

#### (1) Elimination of outliers

To avoid the influence of outliers on the findings of this paper, Eq (1) is regressed again after shrinking the extreme values of the explanatory variable ESI beyond the upper and lower 1% and 10% quartiles, and the results are presented in columns (1) and (2) of Table 3. We find that the coefficient estimates for *ICT*_*s*_ are still significantly positive at the 1% level after removing the outliers and are also similar in magnitude to the baseline regression results. This suggests that the findings of this paper are robust after removing outliers.

**Table 3:** Rejection of outliers and PSM-DID test.

	ESI			
	Baseline regression	1% scaled back	10% scaled back	Cross-sectional PSM
	(1)	(2)	(3)	(4)
*ICTs*	0.6281***	0.6351***	0.4723***	0.6170***
	(0.1890)	(0.1784)	(0.1614)	(0.1890)
*GDP*	0.0196***	0.0144***	-0.0057	0.0199***
	(0.0042)	(0.0041)	(0.0044)	(0.0042)
*FDI*	0.0189**	0.0072	0.0092	0.0185**
	(0.0077)	(0.0084)	(0.0090)	(0.0077)
*finance*	0.3561***	0.2244***	0.0742**	0.3479***
	(0.0484)	(0.0397)	(0.0354)	(0.0484)
*urban*	0.5118	0.5032	0.5059	0.4932
	(0.3926)	(0.4065)	(0.3654)	(0.3934)
*infra*	0.0056	0.0513	0.0256	0.0677
	(0.1117)	(0.1092)	(0.1031)	(0.1132)
*industry*	-0.1958***	-0.2008***	-0.1892***	-0.1999***
	(0.0091)	(0.0093)	(0.0089)	(0.0092)
*er*	-0.0141	-0.0138	-0.0137	-0.0222
	(0.0141)	(0.0135)	(0.0142)	(0.0302)
_*cons*	22.3641***	22.6917***	23.2988***	21.8502***
	(1.4671)	(1.4285)	(1.3471)	(1.4798)
*City fixed effects*	*YES*	*YES*	*YES*	*YES*
*Year fixed effects*	*YES*	*YES*	*YES*	*YES*
N	3696	3696	3696	3668
adj. *R*^2^	0.947	0.949	0.950	0.948

#### (2) Multi-temporal propensity score matching-double difference (PSM-DID) model

Although the establishment of circuit courts is regarded as an exogenous shock, the choice of circuit court establishment is not strictly a natural experiment, and there is still heterogeneity in city characteristics in the empirical analysis. To avoid the problem of selection bias in the assessment of the effect of circuit court establishment, this paper further conducts a robustness check based on a multi-temporal PSM-DID model, using the year-by-year matching method and the panel data transformation method to match propensity scores in turn.

The steps are as follows: **①** Set regional GDP, foreign direct investment, financial development level, urbanization, infrastructure development, industrial structure, and environmental regulation as matching variables. **②** Construct a cross-sectional PSM, i.e., directly apply the nearest-neighbor matching method to find the optimal control group that satisfies the common support condition for all cities in the experimental group, and eliminate the non-common support part, to obtain the new data set. **③** Re-estimate the effect of circuit court establishment on the technical complexity of exports at the city level by applying the multi-temporal DID method. Column (4) in Table 3 reports the results of the multi-temporal PSM-DID regression. The results show that the coefficients on *ICT*_*s*_ remain significantly positive and not substantially different from the baseline regression results, indicating to some extent that the effect of the establishment of circuit courts on city-level export technical complexity is robust.

#### (3) Excluding other policy interferences

The implementation of other relevant policies during the period examined in this paper may also have caused bias in the assessment of the effect of the establishment of the circuit courts. In order to control for the effects of other policies on the estimation results, this paper includes the 2008 policy on the exchange of high court presidents between different locations, the IPR model city policy introduced in 2012 and the free trade test zone policy established in 2015 in the baseline regression model, adding dummy variables for the years in which these three policies were implemented in turn. Which are shown in columns (2), (3) and (4) in Table 4. Where *grj* denotes the policy on foreign exchange of high court presidents; *ipd* denotes the policy on intellectual property demonstration cities; and *freetrade* denotes the policy on free trade test zones. And after controlling for the three types of policies, the estimated coefficients of ICTs are still significantly positive, consistent with the results of the benchmark regression, again indicating that the establishment of circuit courts has a significant elevating effect on export technical complexity at the city level and the findings are robust.

**Table 4:** Other tests of policy interference.

	*ESI*			
	Baseline regression	*grj*	*ipd*	*freetrade*
	(1)	(2)	(3)	(4)
*ICTs*	0.6281***	0.6277***	0.6253***	0.5910***
	(0.1890)	(0.1886)	(0.1889)	(0.1865)
*GDP*	0.0196***	0.0194***	0.0211***	0.0146***
	(0.0042)	(0.0042)	(0.0044)	(0.0043)
*FDI*	0.0189**	0.0180**	0.0186**	0.0185**
	(0.0077)	(0.0076)	(0.0077)	(0.0081)
*finance*	0.3561***	0.3555***	0.3563***	0.3527***
	(0.0484)	(0.0484)	(0.0483)	(0.0454)
*urban*	0.5118	0.6664*	0.5064	0.5577
	(0.3926)	(0.3895)	(0.3918)	(0.3909)
*infra*	0.0056	-0.0074	0.0094	-0.0140
	(0.1117)	(0.1119)	(0.1121)	(0.1120)
*industry*	-0.1958***	-0.1971***	-0.1964***	-0.1961***
	(0.0091)	(0.0090)	(0.0092)	(0.0091)
*er*	-0.0141	-0.0156	-0.0139	-0.0151
	(0.0141)	(0.0141)	(0.0141)	(0.0140)
*grj*		0.6013***		
		(0.1395)		
*ipd*			-0.1548	
			(0.1824)	
*freetrade*				1.4579***
				(0.3517)
_*cons*	22.3641***	22.3209***	22.3346***	22.7016***
	(1.4671)	(1.4655)	(1.4683)	(1.4693)
*City fixed effects*	*YES*	*YES*	*YES*	*YES*
*Year fixed effects*	*YES*	*YES*	*YES*	*YES*
*N*	3696	3696	3696	3696
adj. *R*^2^	0.947	0.947	0.947	0.948

#### (4) Synthetic control method

**Fig 3:**
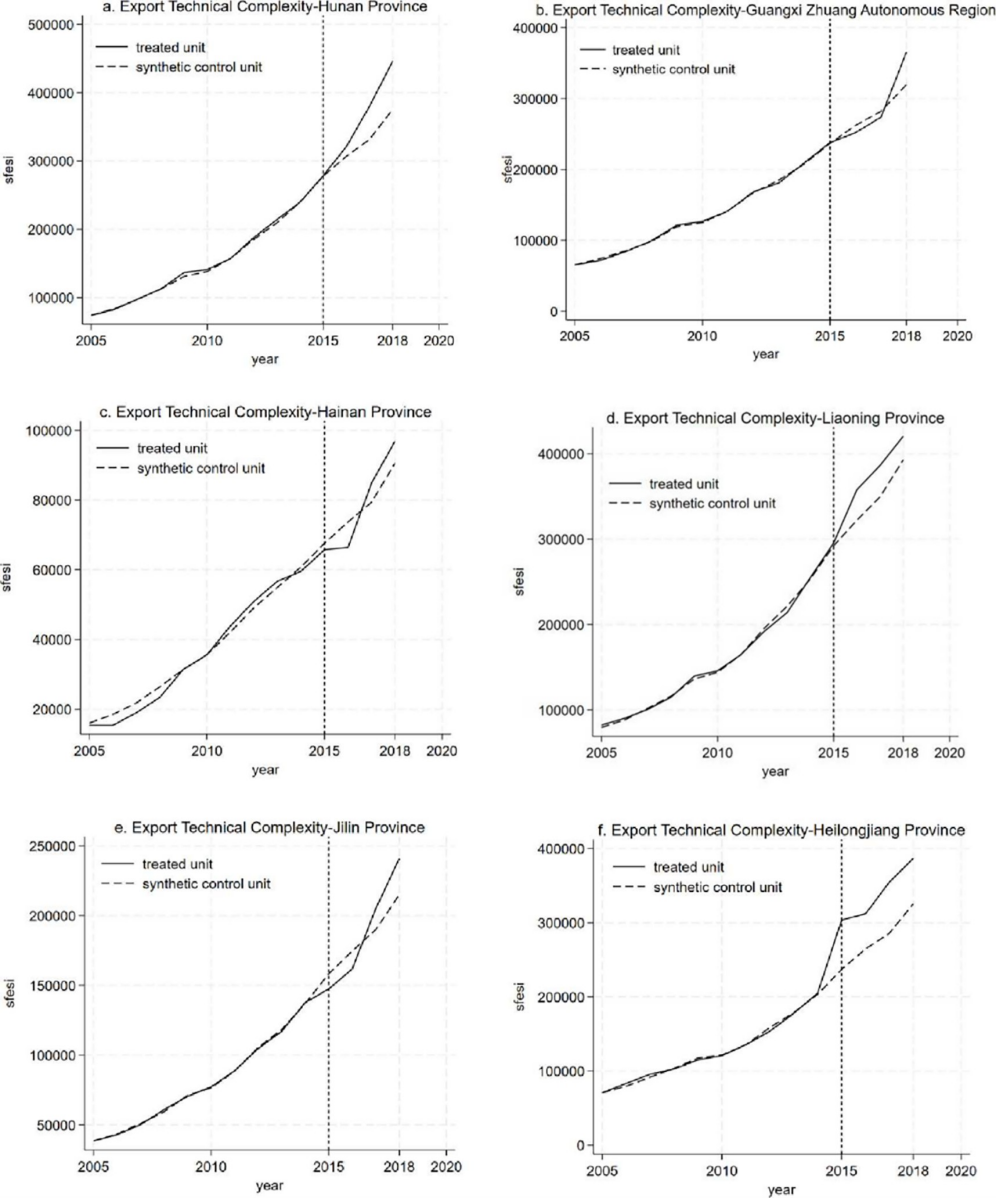
Synthetic control method.

In order to objectively and accurately assess the impact of the establishment of circuit courts on the complexity of technology exports, this study adopts an alternative method proposed by Abadie and Gardeazabal [68] for identifying policy effects—the Synthetic Control Method (SCM), to conduct robustness tests. Given that Guangdong Province is a major player in foreign trade, located in a coastal region and significantly influenced by a series of favorable foreign trade policies, it is excluded from the analysis. Instead, the focus is on the initial six provinces covered by the first batch of circuit courts. Multiple reference groups are weighted to construct a reference group that is entirely similar to the treatment group. The robustness analysis is then carried out. The results are presented in Fig 3.

Fig 3(A)-3(F) consistently demonstrate that, prior to the establishment of circuit courts, the values of the six provinces and their synthetic control counterparts were highly proximate, indicating a well-fitted model. However, following their designation as circuit court regions, the disparities between each province and its synthetic control counterpart markedly widened. The difference between the two represents the impact of circuit court establishment on the complexity of technology exports. Specifically, Guangxi Zhuang Autonomous Region, Hainan Province, and Jilin Province exhibited a delayed policy effect. Nevertheless, the final export technology complexity of their synthetic control counterparts was consistently lower than that of provinces with circuit courts, indicating a discernible positive effect of circuit court establishment on the enhancement of export technology complexity. This suggests that judicial improvements can significantly promote the elevation of export technology complexity.

### (v) Heterogeneity analysis

#### (1) Heterogeneity in the administrative hierarchy of cities

Heterogeneity analysis was conducted according to administrative rank differences. This paper assigns a dummy variable of 1 to the rank of the provincial capital city, special economic zone city, and planned city in the experimental group and 0 to the other cities, and then regresses them separately with the control group to explore whether the effect of the establishment of circuit courts shows significant heterogeneity according to the rank of cities. The results presented in columns (1) and (2) of Table 5 reveal notable patterns regarding the coefficients of *ICT*_*s*_ in various city categories. Specifically, the coefficients for provincial capital cities, economic special zone cities, and planned single-city municipalities are significantly positive at the 1% significance level, with substantial estimated values. Conversely, for non-provincial capital cities, economic special zone cities, and planned single-city municipalities, the coefficients for *ICT*_*s*_ are significantly positive at the 10% significance level, yet the estimated values are comparatively smaller, indicating that the effect of circuit court establishment on the administrative rank of the stronger effect of the establishment of circuit courts on the technical complexity of exports in higher cities. The main reason is that: in provincial capitals, special economic zones, and planned cities with comparative advantages in economic strength, infrastructure development, and foreign investment, commodities and factors can flow and cluster freely, and the clustering effect of factor resources can help enterprises save production costs, transaction costs and search costs, thus further releasing the effect of the establishment of circuit courts. Whereas general cities are weaker than the above-mentioned cities in terms of economic strength, factor concentration, and business environment, thus limiting to a certain extent the effect of the establishment of circuit courts.

**Table 5:** Heterogeneity in administrative class and location of cities.

	*ESI*					
	(1)	(2)	(3)	(4)	(5)	(6)
*ICTs*	2.2006***	0.3606*	2.0928***	0.2740	2.0265***	0.2762
	(0.4031)	(0.1952)	(0.3729)	(0.1926)	(0.3656)	(0.1927)
*GDP*	0.0219***	0.0155***	0.0236***	0.0225***	0.0239***	0.0222***
	(0.0047)	(0.0051)	(0.0055)	(0.0043)	(0.0055)	(0.0043)
*FDI*	0.0136*	0.0266***	0.0136*	0.0143*	0.0130*	0.0151**
	(0.0072)	(0.0089)	(0.0077)	(0.0073)	(0.0077)	(0.0074)
*finance*	0.3644***	0.3241***	0.3484***	0.3624***	0.3500***	0.3595***
	(0.0466)	(0.0479)	(0.0509)	(0.0505)	(0.0506)	(0.0507)
*urban*	0.9433*	0.5809	1.0420**	0.5581	1.0329**	0.5476
	(0.4931)	(0.3867)	(0.5100)	(0.3837)	(0.5095)	(0.3836)
*infra*	-0.0441	0.0298	-0.0527	0.0315	-0.0512	0.0298
	(0.1156)	(0.1124)	(0.1155)	(0.1125)	(0.1154)	(0.1126)
*industry*	-0.1765***	-0.1961***	-0.1781***	-0.1937***	-0.1776***	-0.1943***
	(0.0096)	(0.0091)	(0.0096)	(0.0092)	(0.0096)	(0.0092)
*er*	-0.0191	-0.0157	-0.0216	-0.0127	-0.0180	-0.0140
	(0.0128)	(0.0137)	(0.0132)	(0.0133)	(0.0126)	(0.0133)
_*cons*	21.0695***	22.0790***	21.2845***	21.7002***	21.2432***	21.7600***
	(1.5440)	(1.4796)	(1.5427)	(1.4810)	(1.5431)	(1.4818)
*City fixed effects*	*YES*	*YES*	*YES*	*YES*	*YES*	*YES*
*Year fixed effects*	*YES*	*YES*	*YES*	*YES*	*YES*	*YES*
*N*	3412	3644	3428	3628	3430	3626
adj. *R*^2^	0.946	0.944	0.942	0.947	0.943	0.947

#### (2) Heterogeneity of urban locational characteristics

Heterogeneity analysis was carried out according to the location characteristics of the cities. Firstly, the median distance from the city to the coastline and the port were taken separately, and if the distance from the city to the coastline and the port in the experimental group was greater than the median, the dummy variable was assigned a value of 1, and vice versa, and secondly regressions were conducted separately with the control group to explore whether the effect of the establishment of circuit courts showed significant heterogeneity according to different locational characteristics. Columns (3), (4), (5), and (6) of Table 5 report the final empirical results for different district characteristics respectively. The results show that the coefficients of ICTs for distance from the city to coastline and distance from the city to port greater than the median in columns (3) and (5) are significantly positive at the 1% level, and the estimated coefficients are relatively large, indicating that the establishment of circuit courts has a more pronounced effect on the technological complexity of exports in mid-western cities. The main reason is that: the eastern coastal cities have obvious resource and institutional advantages, but the priority of rapid development has led to the full release of their policy effects, and the excessive use of judicial policies such as the establishment of circuit courts to stimulate trade could instead lead to a mismatch of resources. The opposite is true for cities in the central and western regions, whose urban pilot policy effects have not yet been fully released, and to a certain extent, pilots such as the establishment of circuit courts still have strong scope for play in the region.

#### (3) Rule of law level

In this paper, the heterogeneity of local rule of law levels is measured by the economic case settlement rate, i.e., the ratio of the number of cases settled to the number of cases received in each province. The dummy variable is assigned a value of 1 if the economic case settlement rate in the experimental group is greater than the median, indicating an advanced level of local rule of law, and 0 if the opposite is true. Columns (1) and (2) of Table 6 report the empirical results for local rule of law levels greater than the median and less than the median, respectively. The results show that the coefficient on ICTs in column (2) is significantly positive at the 1% level for cities lagging in rule of law compared to column (1), and the estimated coefficient is relatively larger, indicating that the establishment of circuit courts has a stronger effect on the technical complexity of product exports in cities lagging in rule of law. The main reasons for this are, firstly, the positive changes in the rule of law, serving as a proactive measure with decreasing marginal costs, exhibit significant short-term effects. These changes contribute to enhancing enterprise productivity, fostering the upgrading of export product quality, and inducing an elevation in export technological complexity. secondly, there exists a noteworthy disparity in the efficiency of judicial system execution among different provinces and cities in China. In regions where the rule of law lags or is imperfect, local government interventions in the judiciary are more prevalent. The phenomena of unreasonable local government supervision are common, leading to a lack of effective protection for the production, investment, and trade activities of non-local enterprises. This results in increased rent-seeking costs for enterprises, fostering a pessimistic market expectation among rent-seekers. Such circumstances hinder optimal economic decision-making, consequently reducing welfare levels [69].

**Table 6:** Heterogeneity of local rule of law levels and business environment.

	*ESI*			
	(1)	(2)	(3)	(4)
*ICTs*	-0.2249	0.9422***	0.5141***	1.7045***
	(0.2908)	(0.2043)	(0.1871)	(0.5761)
*GDP*	0.0197***	0.0259***	0.0233***	0.0211***
	(0.0052)	(0.0042)	(0.0043)	(0.0052)
*FDI*	0.0136*	0.0169**	0.0196**	0.0124
	(0.0079)	(0.0075)	(0.0076)	(0.0078)
*finance*	0.4372***	0.2844***	0.2935***	0.3998***
	(0.0558)	(0.0418)	(0.0435)	(0.0523)
*urban*	0.7693*	0.8431**	0.4812	1.0858**
	(0.4460)	(0.4135)	(0.3858)	(0.5166)
*infra*	-0.0225	0.0267	0.0283	-0.0383
	(0.1161)	(0.1108)	(0.1120)	(0.1162)
*industry*	-0.1771***	-0.1904***	-0.1952***	-0.1762***
	(0.0097)	(0.0091)	(0.0091)	(0.0096)
*er*	-0.0168	-0.0226	-0.0142	-0.0196
	(0.0125)	(0.0149)	(0.0138)	(0.0129)
_*cons*	20.7553***	21.6683***	22.1670***	20.7430***
	(1.5468)	(1.4623)	(1.4784)	(1.5465)
*City fixed effects*	*YES*	*YES*	*YES*	*YES*
*Year fixed effects*	*YES*	*YES*	*YES*	*YES*
*N*	3450	3606	3666	3390
adj. *R*^2^	0.943	0.947	0.946	0.943

The establishment of circuit courts effectively eliminates market segmentation, clarifying the boundaries between government and the market. This, in turn, aids in harnessing the dominant role of the market, optimizing the business environment, and fostering enterprise innovation. Overall, these developments contribute to the enhancement of export technological complexity.

#### (4) Political and business environment

This paper uses data on corruption at the provincial level to measure the local business environment and to analyze the heterogeneity of the business environment. The dummy variable is assigned a value of 1 if the data on corruption in the experimental group is greater than the median and 0 if the data on corruption in the experimental group is greater than the median, and then regressed against the control group to explore whether the effect of the establishment of circuit courts is significantly heterogeneous depending on the business environment. Columns (3) and (4) of Table 6 report the empirical results on the strengths and weaknesses of the political and business environment respectively. The results show that the establishment of a city pilot circuit court has a significant positive policy effect regardless of the political and business environment. However, the estimated coefficients of ICTs in column (4) are relatively larger for cities with a better business environment compared to column (3), indicating that the establishment of circuit courts has a stronger effect on the technological sophistication of exports in cities with a better business environment. The main reasons for this are: in the short term, the effect of the establishment of a circuit court does not have a significant effect on the positive change in marginal costs; and the establishment of a circuit court does not immediately change the long-standing and solidified relationship between government and business within the city; and the favoritism of government officials and the illegal profits of enterprises require long-term legal and social supervision. So the effect of the establishment of a circuit court in a city with a poor political and business environment is relatively weak. The intrinsic meaning of the difficulty of starting everything and the long-term need to reverse negative situations is well documented. This corresponds to the conclusion in the previous parallel trend chart that the policy effect of the establishment of circuit courts in the first year is present but not significant; conversely, the effect of the establishment of circuit courts in cities with a better business environment has a significant effect on the positive change in diminishing marginal costs, so that the establishment of circuit courts has a stronger effect on the increase in the technical sophistication of product exports in cities with a better business environment.

### (vi) Analysis of impact mechanisms

The previous section of the theoretical analysis describes the relationship between local protectionism and firm productivity. Other things being equal, a favorable institutional environment can have a two-sided effect on different types of firms, and the combined effect of the two needs to be further tested. Therefore, this paper constructs the following moderating effect model:

$${ESI}_{it}=\alpha +\beta_{1}{ICTs}_{it}+\beta_{2}{ins}_{it}+\beta_{3}{ICTs}_{it}\times {ins}_{it}+\beta_{4}X_{it}+\mu_{t}+\gamma_{i}+\varepsilon_{it}$$ (5)

Where *ins*_*it*_ is the mechanism variable institutional environment, a significant and positive *β*_3_ indicates a more pronounced productivity-enhancing effect due to the exit of low-productivity firms, while a significant and negative *β*_3_ indicates a more pronounced productivity-reducing effect due to the increased cost of compliance by high-productivity firms. Here, Fan et al.’s marketization index [70] is used to measure the institutional environment, which is obtained by weighting six sub-indicators: government-market relationship score, non-state economic development score, product market development score, factor market development score, intermediary organization development, and legal score, to verify that the institutional environment has an impact on the policy effect of the circuit court city pilot. Column (3) of Table 7 tests the effect of the marketization index on the policy effect of the urban pilot of circuit courts, and the results show that the estimated coefficient of ICTs is 2.4409, which is significantly positive at the 1% level. This indicates that the establishment of circuit courts facilitated the increase in export technology sophistication under the combined effect of market-based development and policy guidance. In contrast, the coefficient on the interaction term between institutional environment and circuit court is significantly negative at the 1% level, suggesting a more pronounced productivity-reducing effect due to higher compliance costs for high-productivity firms. Thus, the conclusion that the establishment of local circuit courts is conducive to driving up the overall product export technical complexity is further confirmed. But with due consideration of regional institutional environment heterogeneity, local governments will need to consider the policy effects of the establishment of circuit courts from multiple perspectives once the productivity-enhancing effects of low-productivity firms exiting the market are lower than the productivity-reducing effects of higher-productivity firms’ increased compliance costs.

**Table 7:** Mechanisms of influence of the institutional environment.

	*ESI*		
	(1)	(2)	(3)
ICTs	2.2336***	15.3662***	2.4409***
	(0.7938)	(1.5496)	(0.6124)
*ins*	-0.5039***	0.4472***	-0.2453***
	(0.0971)	(0.0737)	(0.0818)
ICTs×*ins*	-0.2015***	-0.8998***	-0.2333***
	(0.0952)	(0.1977)	(0.0732)
*GDP*		0.1094***	0.0240***
		(0.0082)	(0.0042)
*FDI*		-0.1574***	0.0181**
		(0.0153)	(0.0075)
*finance*		0.2658***	0.3387***
		(0.0392)	(0.0477)
*urban*		1.0085*	0.3761
		(0.5285)	(0.3949)
*infra*		1.5230***	0.0036
		(0.1464)	(0.1124)
*industry*		-0.2123***	-0.1897***
		(0.0097)	(0.0094)
*er*		0.0933	-0.0122
		(0.0955)	(0.0144)
_*cons*	18.494***	-0.1616	23.7986***
	(0.6484)	(1.7726)	(1.5404)
*City fixed effects*	*YES*	*NO*	*YES*
*Year fixed effects*	*YES*	*NO*	*YES*
*N*	3696	3696	3696
adj. *R*^2^	0.9304	0.4913	0.948

## VI. Conclusions and policy recommendations

Against the backdrop of rising global economic uncertainty, building a strong trade nation is an important issue in achieving high-quality economic development in China’s new development landscape, and an important step in promoting a high level of openness, while improving the technical complexity of exports is a necessary step in promoting the building of a strong trade nation. So, this paper explores how effectively elevating the overall export technological complexity through promoting judicial independence.

This paper first constructs a theoretical framework on the impact of local judicial improvements on domestic market integration and export technical complexity, and then systematically evaluates the economic effects of the pilot policy using a quasi-natural experiment on the establishment of circuit courts and a multi-temporal DID model using panel data of 264 prefecture-level cities from 2005–2018. The findings reveal, firstly, the establishment of circuit courts significantly increased the technical complexity of exports in the pilot cities during the sample period, and this finding was held after a series of robustness tests including parallel trend tests, placebo tests, propensity score matching models, and the exclusion of other factors. Secondly, the heterogeneity analysis found that the heterogeneity of the impact of the establishment of circuit courts on the technical complexity of exporting products in the pilot cities was related to the administrative level, geographical location, and the level of rule of law, i.e. the effect of the establishment of circuit courts on the technical complexity of exporting was more obvious in the group of cities with higher administrative level, better location advantage and less advanced rule of law. Thirdly, mechanism analysis shows that a superior institutional environment can have a two-sided effect on different types of firms. Overall, however, the productivity-enhancing effect of low-productivity firms exiting the market is lower than the productivity-reducing effect of higher-productivity firms’ increased compliance costs, which in turn weakens the positive promotional effect of the circuit court.

Based on the theoretical and empirical findings, this paper proposes the following policy recommendations:

Firstly, deepen the Impact of Circuit Courts: The government should steadfastly advance judicial system reforms by further refining the institutional design and arrangement of circuit courts. Given the current status of China’s legal system construction, which still requires improvement, addressing issues such as local protectionism and market segmentation is crucial. The study indicates that the judicial independence brought about by the establishment of circuit courts can enhance enterprise productivity and elevate export technological complexity, contributing to the optimization of trade structures and the construction of a trade powerhouse. Given that the current pilot policies are in their early stages with limited coverage and penetration, it is imperative for the government to deepen the impact of circuit courts by refining their institutional design, fully leveraging their policy effects, and harnessing the potential of legal governance to enhance product export quality and overall socio-economic development.

Secondly, account for regional heterogeneity in policy effects: Acknowledge and address regional heterogeneity in the effects of circuit court policies. The policy design of circuit courts should consider regional differences and emphasize the necessity of balanced regional coordination. Tailored policy measures, aligned with the market conditions, business environment, and characteristics of enterprises in different regions, should be formulated to ensure the effectiveness and compliance of circuit court policies. Attention should be paid to regional coordination and progress monitoring to avoid potential disparities in export technological complexity and economic development arising from differences in the effects of judicial system reform.

Thirdly, consider the impact of local policies and market heterogeneity: While policies such as judge cross-regional exchanges, intellectual property protection, and free trade zone pilot policies do not interfere with the functioning of circuit courts, the study suggests that the impact of these policies on enterprises varies depending on the completeness of the institutional environment. Therefore, local governments in pilot areas should consider the heterogeneity of institutional environments, improve contract environments, coordinate different institutions, and explore legal policies more suitable for superior institutional environments. Measures such as implementing and publicizing lists of untrustworthy individuals and advancing the construction of a social credit system in the era of big data can enhance judicial perfection, improve the quality of exported products, raise regional export technological complexity, promote industrial structure upgrading, and facilitate high-quality economic development.

Finally, address market entry barriers: given that the positive effect of circuit courts is weakened by the lower productivity gains from the exit of low-productivity enterprises compared to the productivity losses incurred by high-productivity enterprises due to increased legal compliance costs, adjustments to market entry barriers are recommended. In the context of a well-established legal system, tailored measures should be taken to adjust market entry barriers for enterprises, such as providing targeted support to small and medium-sized enterprises, advancing tax reductions and fee reductions, lowering institutional transaction costs, and optimizing the business environment. These measures aim to stimulate the vitality of market entities, strengthen the positive promotion of circuit courts, create an independent and just judicial environment, foster a favorable business atmosphere, promote the development of market integration, and elevate product quality, thereby contributing to the enhancement of export technological complexity and the stabilization of economic growth.

## References

[1] Hausmann D, Hwang J, Rodrikd D. What you export matters[J]. Journal of Economic Growth. 2007; 12(1):1–25.

[2] Ma SZ, Ren WW, Wu GJ. Characteristics of a country’s agricultural trade network and its impact on the division of labor in global value chains—based on a social network analysis perspective[J]. Journal of Management World. 2016; (03):60–72.

[3] Mao QL, Fang LH. Innovation drive and export technological complexity of Chinese manufacturing firms[J]. Forum of World Economics & Politics. 2018; (02):1–24.

[4] Sheng B, Mao QL. Does Import Trade Liberalization Affect Chinese Manufacturing Export Technological Sophistication[J]. The Journal of World Economy. 2017; 40(12):52–75.

[5] Yuan C, Geng CX, Cong HY, Xiao TS. Regional Judicial Quality and Industrial Structure Upgrading: Evidence from the Establishment of Circuit Courts[J]. Economic Research Journal. 2023; 58(09):171–189.

[6] Lei N, Lang LH. The Impact of the Domestic Market Integration on the Export Technical Sophistication and Its Mechanism[J]. Statistical Research. 2020; 37(02):52–64.

[7] Gong T. ‘Dependent Judiciary and Unaccountable Judges: Judicial Corruption in Contemporary China.’ China Review. 2004; 4(2),33–54.

[8] Melitz MJ. The Impact of Trade on Intra-Industry Real locations and Aggregate Industry Productivity. EconMetrica. 2003;71(6),1695–1725.

[9] Lv Y, Lv YL, Gao Y. Comparative advantage of intermediate goods market segmentation and manufacturing exports-based on the perspective of global value chain. Industrial Economics Research. 2017; (5),51–61.

[10] Groenewold N, Lee G, Chen A. Regional output spillovers in China: estimates from a VAR Model. Papers in Regional Science. 2007; (86),101–122.

[11] Venables AJ. Productivity in cities: self-selection and sorting. Journal of Economic Geography. 2011; 11(2),241–251.

[12] Yuan X, Wu LH, Zhang P. Integrated development of Yangtze River Economic Belt and R&D efficiency of high-tech industries. Research in Quantitative Economics and Technology Economics. 2019; 36(4),46–61.

[13] Sheng B, Mao QL. Trade openness, domestic market integration and inter-provincial economic growth in China: 1985–2008. World Economy. 2011; (11),44–66.

[14] Poncet S. Measuring Chinese Domestic and International Integration. China Economic Review. 2003; 14(1),1–21.

[15] Hou KW, David T, Robinson. Industry Concentration and Average Stock Returns. Journal of Finance. 2006; 62(4),1927–1956.

[16] Zhang J, Zhang PL, Huang TY. Has market segmentation boosted Chinese firms’ exports. Economic Research. 2010; (8),29–41.

[17] Shi CK, Liang HJ. Administrative monopoly, market entry cost and export productivity paradox: An empirical analysis based on provincial dynamic panel data of Chinese industries. Economic and Management Studies. 2013; (9),28–37.

[18] Bernard AB, Stephen J, Redding SJ, Schott PK. Comparative Advantage and Heterogeneous Firms, The Review of Economic Studies. 2007; 74(1),31–66.

[19] Mao QL. Domestic market integration and China’s export technology level: A theoretical and empirical study based on financial development perspective. World Economic Journal. 2012; (3),14–40.

[20] Kee HL, Tang HW. "Domestic Value Added in Exports: Theory and Firm Evidence from China," American Economic Review, American Economic Association. 2016; 106(6),1402–1436.

[21] Shen DY, Cao SB, Shi XZ. The construction of judicial power in China under the perspective of national governance. China Social Science. 2015; (3),39–57+206.

[22] Bai CE, Hsieh CT, Song Z. Special deals with Chinese characteristics. NBER Macroeconomics Annual. 2019; 34,341–379.

[23] Head K, Mayer T. Brands in Motion: How frictions shape multinational production. American Economic Review. 2019; 109(9),3073–3124.

[24] Nunn N. ‘Relationship-specificity, incomplete contracts, and the pattern of trade,’ The Quarterly Journal of Economics. 2007; 122(2),569–600.

[25] Ernest Liu, Lu Y Peng WW, Wang SD. “Judicial Independence, Local Protectionism, and Economic Integration: Evidence from China.” NBER Working Paper No. 30432. 2022; doi: 10.3386/w30432

[26] Donaldson D. “The gains from market integration,” economics. 2015; 7(1),619–647.

[27] Zhou L.A. The administrative subcontract: Significance, relevance and implications for intergovernmental relations in China. Chinese Journal of Sociology. 2016; 2(1),34–74.

[28] Young A. “The razor’s edge: Distortions and incremental reform in the People’s Republic of China,” The Quarterly Journal of Economics. 2000; 115(4),1091–1135.

[29] Bai YJ, Du YY, Tao ZT, Tong SY. “Local protectionism and regional specialization: evidence from China’s industries,” Journal of international economics. 2004; 63(2),397–417.

[30] Barwick PJ, Cao SM, Li SJ. “Local protectionism, market structure, and social welfare: China’s automobile market,” American Economic Journal: Economic Policy. 2021; 13(4),112–51.

[31] Bao ZY, Huang DF, Lin C. Can Artificial Intelligence Improve Gender Equality? Evidence from a Natural Experiment (August 27, 2022). Available at SSRN: https://ssrn.com/abstract=4202239 or doi: 10.2139/ssrn.4202239

[32] Acemoglu D, Robinson JA. Why nations fail: The origins of power, prosperity, and poverty, Currency. 2012.

[33] LaPorta R, López-de-Silanes Florencio, Pop-Eleches Cristian, Andrei S. “Judicial Checks and Balances”, Journal of Political Economy. 2004; 112(2),445–470.

[34] Chakraborty P. Judicial quality and regional firm performance: The case of Indian state. Journal of Comparative Economics. 2016; 44(4),902–918.

[35] Chen G, Li S. Judicial Independence and Market Fragmentation——Evidence from the Geographical Rotation of Judges in China[J]. Economic Research Journal. 2013; 48(09):30–42+70.

[36] Cao CF, Chen LL, Zhang TT. "In the name of the law": Increased judicial independence and corporate violations. Financial Research. 2017; (5),191–206.

[37] Yu YC, Chu HL, Wei J. Market segmentation in the context of judicial administrativeization—a model of intertemporal cooperative choice. China Economic Issues. 2020; (4),122–136.

[38] Huang J, Chen XY, Zhao Y, Hu DQ. Judicial improvement and corporate investment-an empirical study based on the establishment of circuit courts in China. Economics (Quarterly). 2021; 21(5),1521–1544.

[39] Kuznets, Dai R, Yi C. Modern Economic Growth. Beijing Academy of Economics Press. 1989.

[40] Chen M, Li N, Zheng L, Huang D, Wu B. Dynamic correlation of market connectivity, risk spillover and abnormal volatility in stock price[J]. Physica A: Statistical Mechanics and its Applications. 2022; 587.

[41] Schott PK. “The Relative Sophistication of Chinese Exports.”Economic Policy. 2008; 23(53),5–49.

[42] Acemoglu D, Restrepo P. Robots and Jobs: Evidence from US Labor Markets. Journal of Political Economy. 2020; 128(6),2188–2244.

[43] Meon PG, Sekkat K. Institutional quality and trade: Which institutions? Which trade? Economic Inquiry. 2008; 46(2),227–240.

[44] Zhu FL, Zhao SQ. Institutional quality, international R&D spillovers and technological complexity of service exports-an empirical study based on cross-country panel data. Explorations in Economic Issues. 2018; (10),151–162.

[45] Hudson J, Minea A. Innovation, Intellectual Property Rights, and Economic Development: A Unified Empirical Investigation. World Development. 2013; 46(2),66–78.

[46] Berkowitz D, Moenius J, Pistor K. Trade, Law, and Product Complexity. The Review of Economics and Statistics. 2006; 88(2),363–373.

[47] Branstetter L, Fisman R, Foley CF, Saggi K. “Does Intellectual Property Rights Reform Spur Industrial Development” Journal of Development Economics. 2011; 83(1),27–36.

[48] Yang LY, Wang Jun. Has IPR protection increased the technological complexity of China’s exports? China Economic Issues. 2015; (3),97–108.

[49] Tao T, Fan KX. Administrative approval reform, firm product decisions and export technology complexity. Journal of Wuhan University (Philosophy and Social Science Edition). 2022; 75(4),114–129.

[50] Chen WD. "The focus and outlook of the new round of judicial reform". China Law: Chinese and English. 2015; (1),14–22.

[51] Liu GX, Hu YT. "Circuit Courts: The ’Pioneers’ of Judicial Reform-An Interview with the Presidents of the First and Second Circuit Courts of the Supreme People’s Court", China Law Review. 2015; (1),18.

[52] Zhou Q. Report of the Supreme People’s Court on the comprehensive deepening of judicial reform in the people’s courts[N]. People’s Court Daily,2017-11-02(001).

[53] Chen HP. The effectiveness and improvement of the reform of the "provincial unified management of human and financial resources". People’s Rule of Law. 2018; (Z1),9–11.

[54] Zhang TS, Ginsburg TB. “China’s Turn Toward Law.” 2019.

[55] Yu MJ, Cui XM, Zhang R. Judicial Quality, Incomplete Contract, and Quality of Trade[J]. Journal of Financial Research. 2016; (12):1–16.

[56] La Porta R, Lopez-Silanes F, Vishny, R.W. Law and Finance[J]. Journal of Political Economy, 1998; 106(6): 1113–1155.

[57] Bao YW. Intellectual Property Protection, Technology Introduction and Technology Innovation of Chinese Manufacturing—An Empirical Test Based on Panel Data[J]. Journal of International Trade. 2017; (06):38–49.

[58] Zheng CY. Intellectual Property Protection and Quality Structure of Export—An Empirical Study Based on US Import Data[J]. Journal of International Trade. 2017; (12):3–1.

[59] Dai ZQ, Li ZS, Gao YS. IPR Protection and the Position of Enterprises’ Global Value Chain—From the Perspective of Intermediate Products’ Supply and Demand[J]. Journal of International Trade. 2021; (05):96–108.

[60] Jiang DC, Peng DD. Domestic Intellectual Property Protection and Outward Foreign Direct Investment[J]. Journal of International Trade. 2023; (10):36–52.

[61] Yang QL, Zhang XY. The Reform of Administrative Approval System and the Technical Complexity of Manufacturing Enterprises’ Exportin China[J]. Journal of International Trade. 2022; (02):106–124.

[62] Ji XY, Gu NH. Does the Establishment of Intellectual Property Model Cities Affect Innovation Quality[J]. Journal of Finance and Economics. 2021; 47(05):49–63.

[63] Sheng D, Bu FC, Wang YJ. Stronger Intellectual Property Protection and Chinese Export Product Quality Upgrading—Evidence from the Establishment of Municipal Patent Agency[J]. Journal of International Trade. 2023; (04):107–123.

[64] Duan F, Zhu JS, Zhong TL, Tang C. International Cooperation on Intellectual Property Rights and Corporate Exports: A Study Based on “Patent Prosecution Highway” Agreements[J]. The Journal of World Economy. 2022; 45(06):32–59.

[65] An JF, Armitage S, Hou WX, Liu XD. Do checks on bureaucrats improve firm value? Evidence from a natural experiment[J]. Accounting & Finance. 2020; (60): 4812–4844.

[66] Baldwin R, Harrigan J. Zeros, Quality and Space: Trade Theory and Trade Evidence[J]. American Economic Journal: Microeconomic. 2011; 3(2): 60–88.

[67] Jeanneny SG, Hua P, Ling Z. Financial Development, Economic Efficiency and Productivity Growth: Evidence from China [J]. Developing Economics. 2006; 44(1),27–52.

[68] Abadie A, Gardeazabal J. The Economic Costs of Conflict: A Case Study of the Basque Country[J]. American Economic Review. 2003; 93(1): 113–132.

[69] Guo S, An J. Does terrorism make people pessimistic? Evidence from a natural experiment, Journal of Development Economics. 2022; 155, 102817.

[70] Fan G, Wang XL, Ma GR. Contribution of Marketization to China’s Economic Growth[J]. Economic Research Journal. 2011; 46(09): 4–16.

